# Supplementary material for: Semantic influences on object detection: Drift diffusion modeling provides insights regarding mechanism
Source: PLoS Comput Biol. 2025 Jun 11;21(6):e1012269. doi: 10.1371/journal.pcbi.1012269 (PMC12194206; doi:10.1371/journal.pcbi.1012269)
Supplement: S1 Text — (DOCX) [file pcbi.1012269.s001.docx]

## Correspondence between Bogacz's and Ratcliff's notation

In Bogacz's notation, drift rate is $A$, threshold is ${\pm z}_{Bogacz}$, starting point is $x_{0}$, non-decision time is $T_{0}$, and noise is $c.$

In Ratcliff's notation, drift rate is $v$,

|  | $v = A$ | (1), |
| --- | --- | --- |

threshold is $a$,

|  | $a = 2*z_{Bogacz}$ | (2), |
| --- | --- | --- |

starting point is $z$,

|  | $z = z_{Bogacz}+x_{0}$ | (3), |
| --- | --- | --- |

non-decision time is $T_{er}$,

|  | $T_{er} = T_{0}$ | (4), |
| --- | --- | --- |

and noise is $s$,

|  | $s = c$ | (5). |
| --- | --- | --- |
