# Supplementary material for: Semantic influences on object detection: Drift diffusion modeling provides insights regarding mechanism
Source: PLoS Comput Biol. 2025 Jun 11;21(6):e1012269. doi: 10.1371/journal.pcbi.1012269 (PMC12194206; doi:10.1371/journal.pcbi.1012269)
Supplement: S2 Text — (DOCX) [file pcbi.1012269.s002.docx]

## Parameter recovery

In this analysis, we simulate behavioral data on our experiment with known parameter values and then fit this simulated data to determine whether the fit parameter values are close to the simulated parameter values. The parameters used for the simulation were the parameters obtained by fitting the full data set. In total, our simulated data set was approximately the same size as our real data set with 103 simulated participants each performing 72 trials in the control and experimental studies; for the control study, we simulated data for 108 participants. For the experimental groups, we simulated data for only 103 participants rather than twice that number since the same model was used for study 1 and study 2. Thus, the simulated data approximated the number of participants tested in the experiment. The fitting procedure for parameter recovery was identical to that used for the real subjects. In Figs A and B below, we plot the relationship between the recovered and simulated parameters for both the control and experimental models.


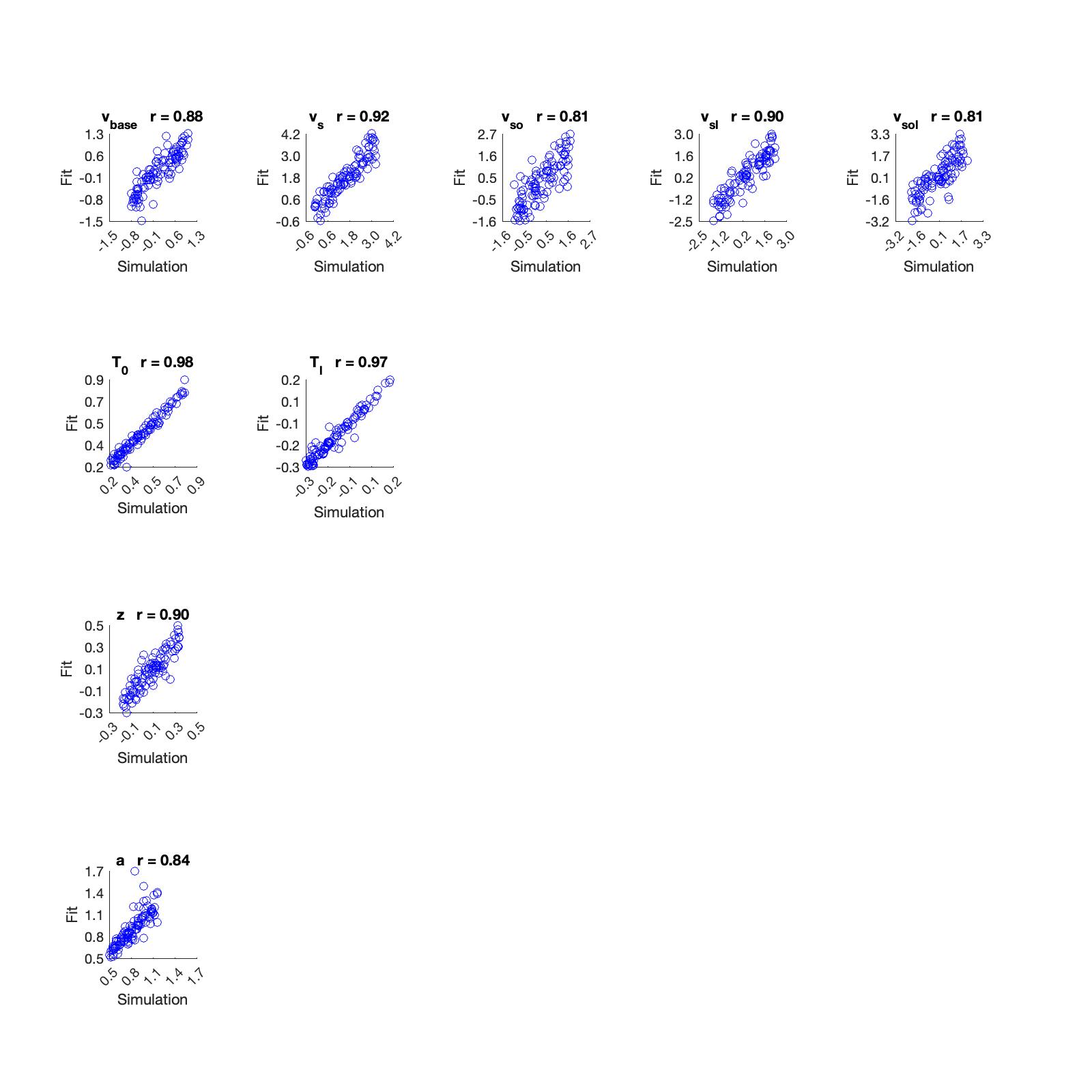


**Fig A** Parameter recovery for the full parameter model of the Experimental conditions with MLE fits. The value on the x axis represents simulation value, and value on y axis represents the value for the fitting process. Each row in the figure represents the components of up to 4 different parameters. Values of r on top of each axis represent the correlation coefficient. Note the scale of the y axis changes across graphs.


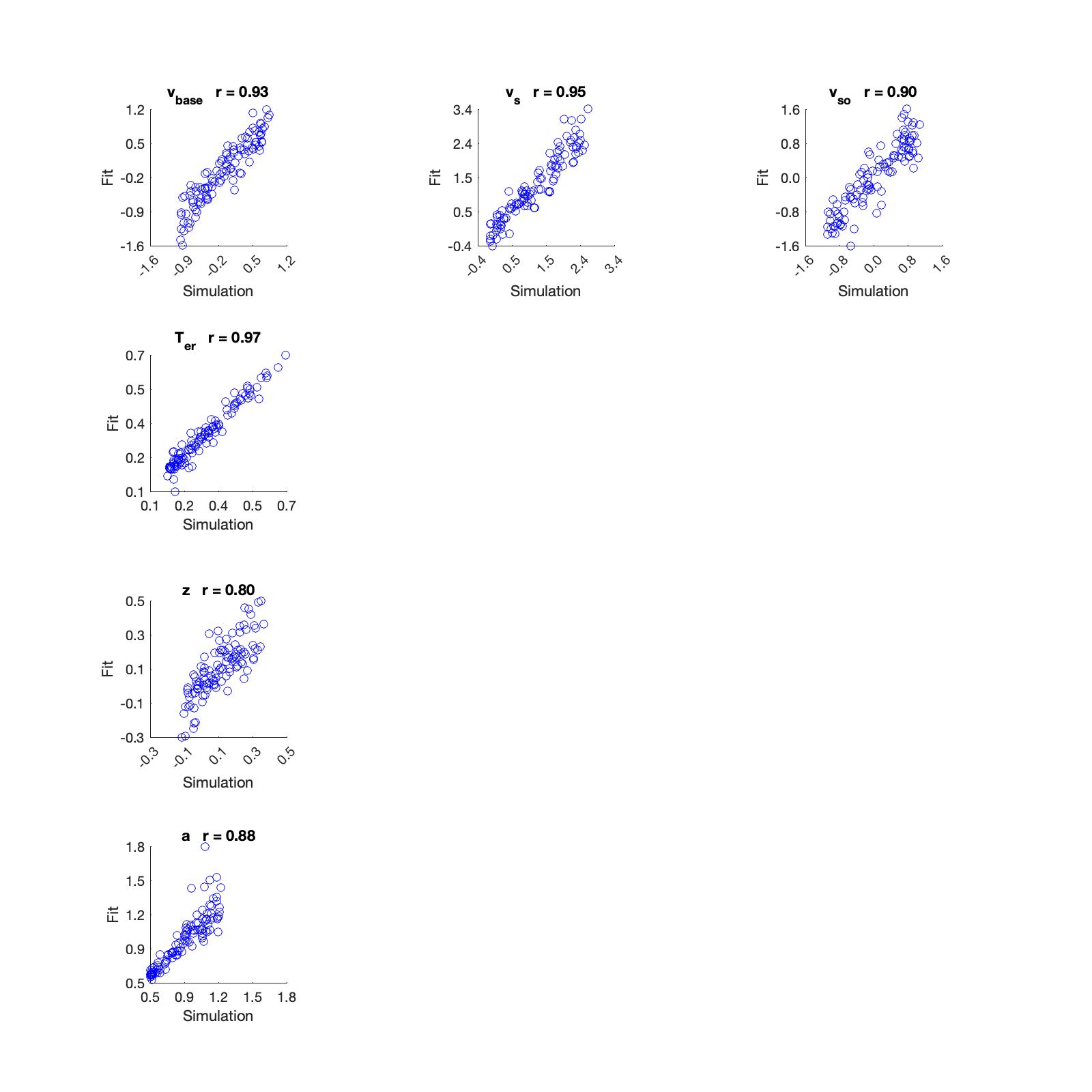


**Fig B** Parameter recovery for the model of the control conditions with MLE fits. Parameter recovery with MLE fits for the full parameter model. Axis and values of r have the same meaning as in Fig S1, the only difference is that we have 6 instead of 9 parameters for the control group.
