# Supplementary material for: Semantic influences on object detection: Drift diffusion modeling provides insights regarding mechanism
Source: PLoS Comput Biol. 2025 Jun 11;21(6):e1012269. doi: 10.1371/journal.pcbi.1012269 (PMC12194206; doi:10.1371/journal.pcbi.1012269)
Supplement: S3 Text — (DOCX) [file pcbi.1012269.s003.docx]

## Quantile analysis


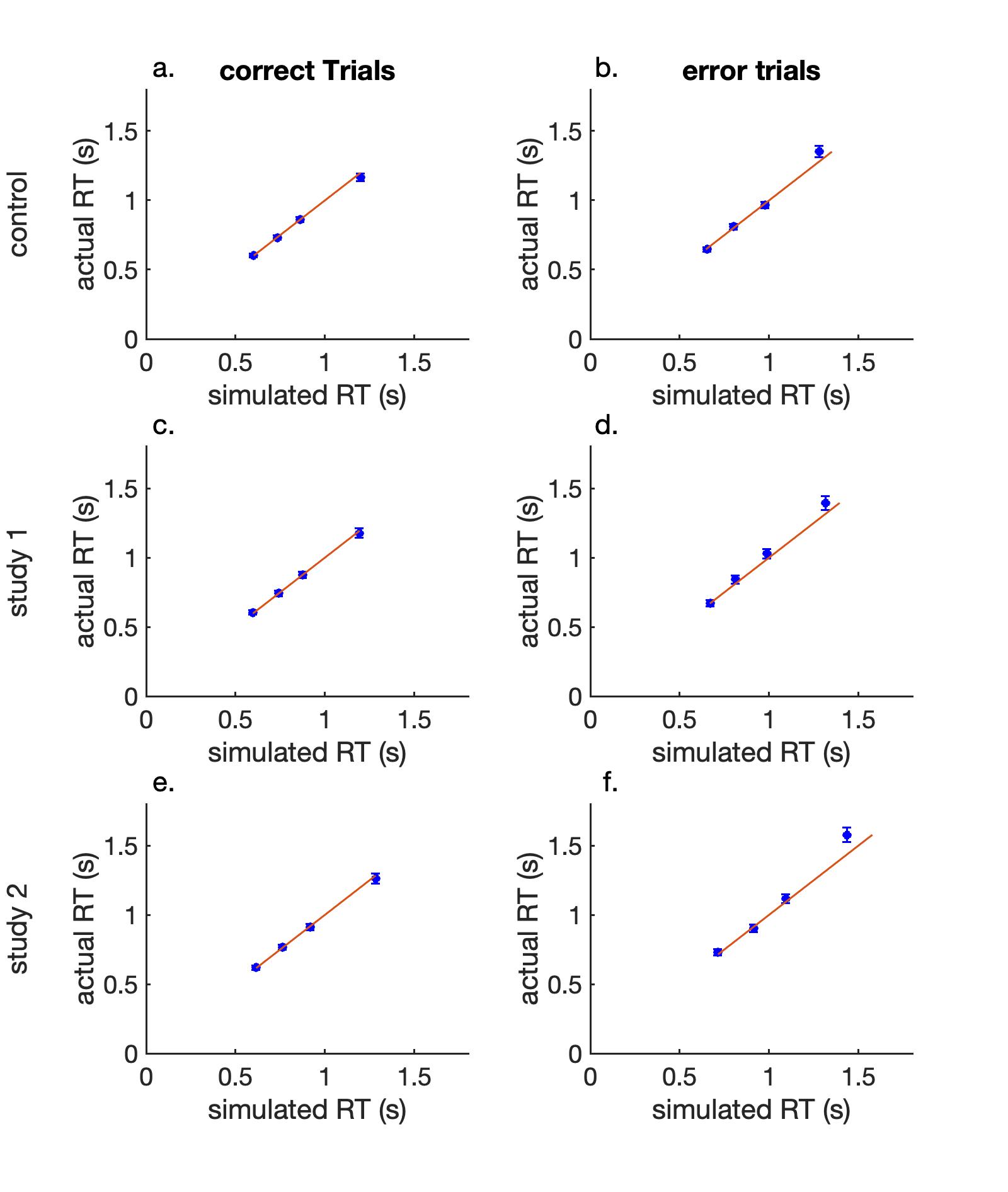


**Fig A** Quantile-quantile plot for the control study. The left column plots are for correct trials; the right column plots are for error trials. The first row represents the control study, the middle row represents study 1, and the bottom row represents study 2. Quantile levels are 24.5%, 49%, 73.5%, 98%, in ascending order from left to right side (the final 2% of trials are excluded). The reaction time of each quantile is computed for each participant first and then averaged over all participants. Error bars are pooled standard error.


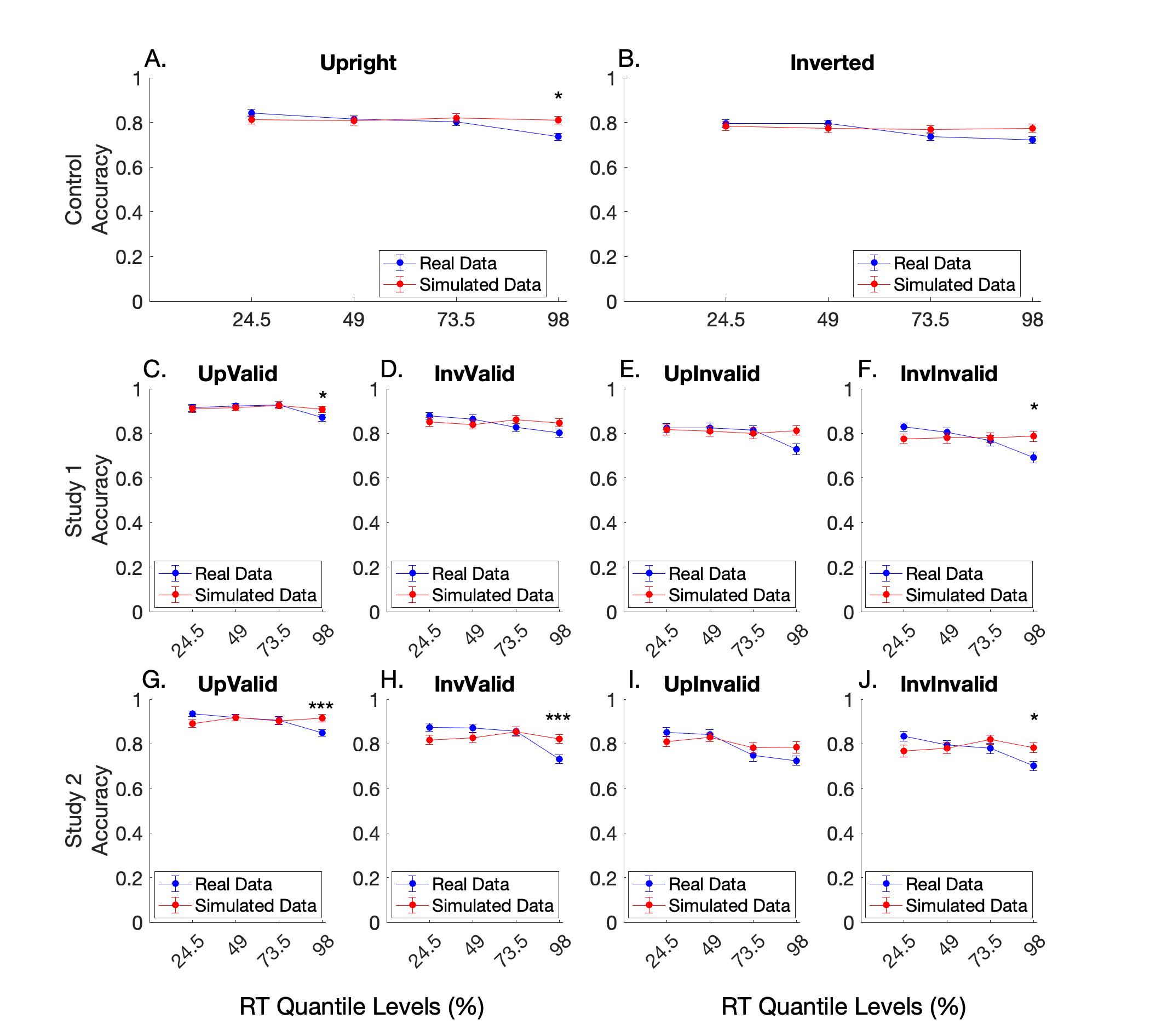


**Fig B** Accuracy plotted against reaction time for actual and simulated data for four quantiles. Quantile levels are 24.5%, 49%, 73.5%, 98%. Averaged accuracy was computed on each quantile for each participant and then averaged over all participants. The first row represents the control study, the middle row represents study 1, and the bottom row represents study 2. In each plot, red line represents the simulation, and the blue line represents actual data. Error bars are pooled standard error. Black asterisks indicate differences between the actual data and the simulated data at the corresponding quantile levels represented along the x-axis. *** indicates *p* < 0.00025, * indicates *p* < 0.0125 after Bonferroni correction. The figure showed a good fit of the model to the data, especially in the first 3 quantiles of the data.
