# Supplementary figures and images for: Semantic influences on object detection: Drift diffusion modeling provides insights regarding mechanism
Source: PLoS Comput Biol. 2025 Jun 11;21(6):e1012269. doi: 10.1371/journal.pcbi.1012269 (PMC12194206; doi:10.1371/journal.pcbi.1012269)

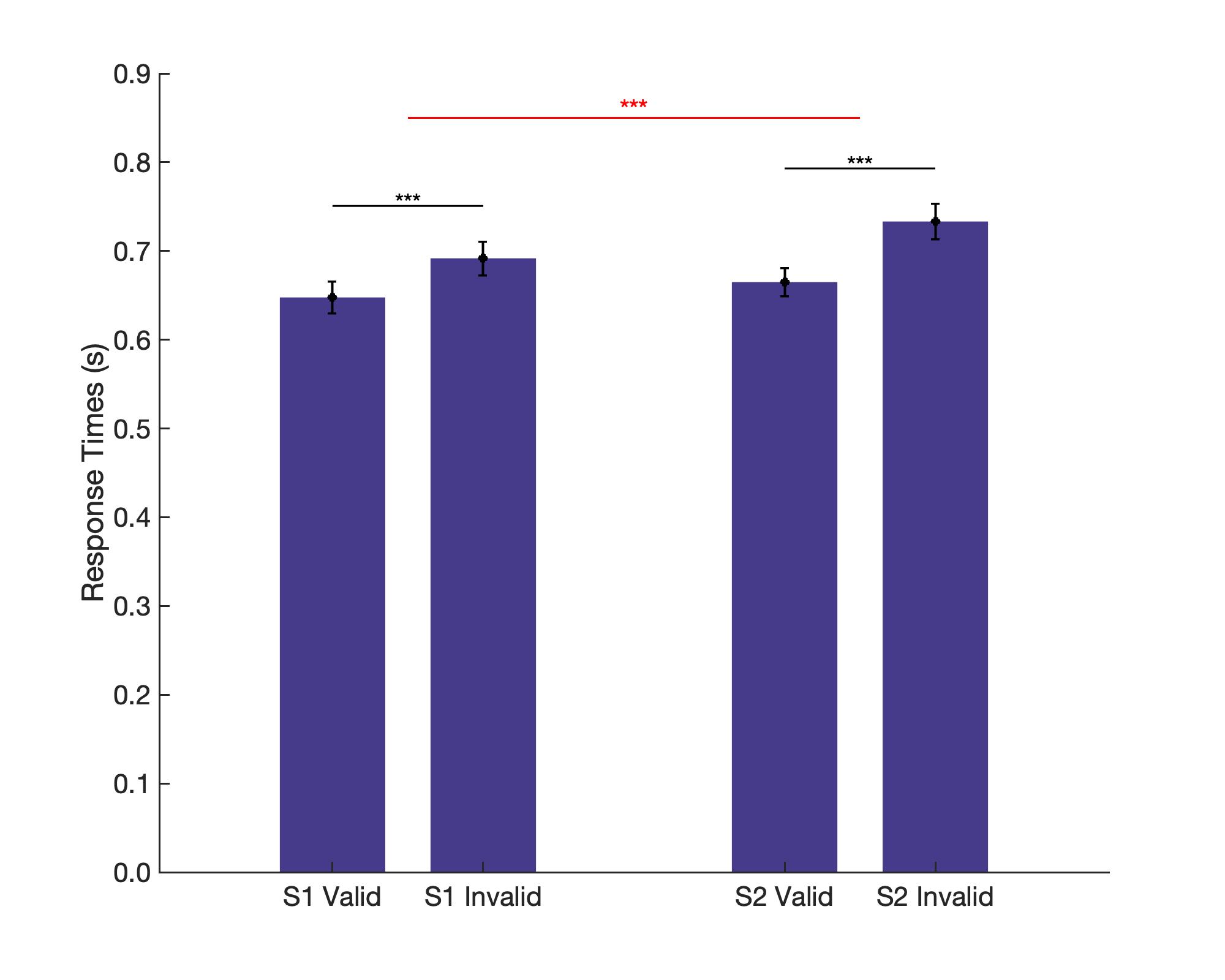

Supplement: S1 Fig — *** indicates p < 0.001. Red asterisks represent interaction between label type and study. (JPG) [file pcbi.1012269.s004.jpg]
