# Supplementary material for: Semantic influences on object detection: Drift diffusion modeling provides insights regarding mechanism
Source: PLoS Comput Biol. 2025 Jun 11;21(6):e1012269. doi: 10.1371/journal.pcbi.1012269 (PMC12194206; doi:10.1371/journal.pcbi.1012269)
Supplement: Attachment — Submitted filename: response_to_reviewer_final.pdf [file pcbi.1012269.s006.pdf]

Response to Reviewers

We greatly appreciate the thoughtful comments and suggestions of the reviewers. In what follows we note each of their comments in blue ink and describe our response in black ink. The revised part from the manuscript is in red ink. Our manuscript is much improved as a consequence of making the changes recommended by the reviewers.

Reviewer 1:

Point 1. I appreciate the care taken to differentiate the label-feedback and semantic network hypotheses, but I was not entirely convinced that the predictions regarding the inversion effect were as clear-cut as the authors made them out to be. On the one hand, I would expect a strong inversion effect for objects encountered in unfamiliar orientations, per the semantic network hypothesis. On the other hand, I would also expect similar disruption under the label-feedback hypothesis since the low-level features activated would presumably be in proportion to the typicality of their presentation. I would expect a feature presented in an unusual context to be more weakly associated with the object representation than I would the same feature presented in a common context. Put another way, are the authors assuming "features" are represented in an entire context-independent manner? I worry this assumption is too strong because it divorces feature representations from potentially useful configural information that would presumably exist at some level of the representational hierarchy.

Response 1:

We appreciate the opportunity to clarify our position regarding the relationship between configural information, feature representations and semantic space, and how this distinction informs our predictions about the inversion effect. We address this question on page 32-33 in a new section, "Label-Feedback Hypothesis versus the Semantic Network Hypothesis".

> **Revised Part (P33-P34):**
>
> Can the Label-Feedback hypothesis be altered to accommodate our results by supposing, for instance, that feature representations are orientation dependent? The classic view in perception research, supported by much research, is that visual features are orientation independent (e.g., Gayet, et al, 2014; Humphreys & Riddoch, 1984), whereas orientation dependence is a hallmark of configuration effects. What about supposing that the Label Feedback hypothesis extends to features in context? We don't see how this would help, as features in context are familiar configurations, which are not low-level representations. In the Label-Feedback hypothesis, feedback from high-level word representations

extends to low-level features of objects (Lupyan & Ward, 2013). Moreover, in other experiments, we have found that that the same features arranged in novel configurations do not operate as object priors, even when sub-configurations comprising familiar parts are intact (e.g., Flowers & Peterson, in review; Peterson, et al, 1991). Finally, our evidence contrary to the Label-Feedback hypothesis extends beyond orientation dependency: Drift rates were lower, and thresholds were higher when labels denoted an object in the same, rather than a different, superordinate-level category as the object in the display. Indeed, in study 1, invalid labels denoting objects in a different superordinate-level category had no effect on either drift rate or thresholds. On the Label-Feedback Hypothesis, the opposite pattern should have been observed because the features of objects in a different superordinate-level category differ more from the features of the objects in the display, and hence, would require more prediction revision which would take longer. These considerations suggest that the Label-Feedback Hypothesis cannot easily be altered to account for our results. The pattern of results is better fit by the Semantic Network Theory of Label effects.

Point 2. Diffusion model description – Although I am highly familiar with the use of these models, I found it difficult to follow the model description on pages 9-11 due to the authors' departure from using conventional notation for the model parameters. For example, drift rate in Ratcliff's model is usually denoted v (not A), response bias is usually denoted z (not x_0), upper and lower boundaries are usually denoted using +a and -a (or a and 0; not, +Z and -Z). This is a relatively minor issue, but it did affect the fluency with which I could parse the model description. This would not be so bad if the authors' notation didn't actively conflict with convention (e.g., as with boundary position and start-point parameters). I think bringing the model description in line with Ratcliff's standard notation would help improve the readability of this section and make it easier for people to relate the current findings to previous work with the DDM. Also, I was initially confused by the description of the c parameter (p.9) and the later reference to "an additional noise parameter" (p.11). I later realized on page 15 that there was only one noise parameter, which was c, but prior to that, I assumed there were two noise parameters (one describing across-trial variability in the drift rate and another describing within-trial accumulation noise; the diffusion coefficient). It would be helpful if the authors clarify on page 9 that c is fixed to 1.

Response 2:

Thanks for pointing this out. We do not intend to confuse our readers. We originally used Bogacz's notation and now switch to Ratcliff's notation. However, since Bogacz's (Bogacz et al., 2006) notation

was used in our original code, we now explain this in the main text and provide the correspondence between the two notations in the supplementary material.

Revised Part (P18):

Note that we use Ratcliff's notation (Ratcliff, & McKoon, 2008) in the current paper, but we want to acknowledge the readers that we used Bogacz's notation (Bogacz et al., 2006) in our original code. The correspondence between Ratcliff's notation and Bogacz's notation is detailed supplementary material.

Revised Part (P10-P11):

Our drift diffusion model (DDM) assumes that, when presented with a bipartite test display, participants make a left/right response by accumulating "evidence" over time in favor of one response or the other. This evidence is assumed to contain both signal – whether the object lies to the left or right of the central border – as well as noise caused e.g., by ambiguous cues and random neuronal responding. Thus, the accumulated evidence, y, evolves according to a drifting random walk with a noise parameter controlling the randomness of the walk and a drift parameter, v, controlling the drift (see Fig 3A). In line with common practice, we set $s = 1$.

Point 3. Model parameterization – The authors mention on page 11 that all four free DDM parameters are estimated across different label conditions. One concern is that this includes decision threshold (denoted by the authors as z). Traditionally, this parameter is only allowed to vary as a function of task-level factors rather than stimulus-level ones because it is regarded as capturing strategic aspects of how a participant is approaching the task (i.e., it controls their speed-accuracy trade-off).

Because the validity of the label is based on its congruency with the stimulus, allowing threshold to vary with label validity requires threshold setting to be highly reactive—occurring when the stimulus is presented, not when the label appears (cf. the authors' description of Equation 7 on page 17). To be clear, determining whether a label is valid or invalid in this task context requires some way of evaluating the correspondence between the stimulus and the label, which logically requires identification of the stimulus ahead of being able to locate it on one side of the display.

Minimally, the threshold parameter needs to be fixed across trials. It can, however, be estimated freely across the three studies, which would still provide important information about how participants process information when labels are drawn from the same vs. different superordinate category compared to having no labels at all.

The authors discuss the matter of threshold not varying based on stimulus conditions with respect to left/right side and upright/inverted orientation (pg.17), but label is regarded differently. In this case, however, I think label has to be treated in the same way because its effects can only be realized in relation to the properties of the stimulus.

Response 3:
Thank you very much for pointing out this important point. We agree that threshold should be fixed across different label conditions. We therefore fixed the threshold across valid and invalid label conditions within experiments. Making this change led to some changes in the results for threshold. Specifically, thresholds were not higher in study 1 than in the control study (see pp. 25). Accordingly, we removed our claim that detection entails semantics even on control trials. Thresholds were significantly higher in study 2 than in the control study, as we reported previously.

Point 4. A similar argument can be made about start-point needing to be fixed across valid/invalid label conditions. Allowing this parameter to vary on a trial-by-trial basis would make sense if the labels were more directly related to the response options (e.g., saying "left" vs. "right"), in which case x0 would make sense as capturing response bias. Here, however, the lack of any direct mapping from label-stimulus congruency to the left/right response makes it questionable as to why/how start-point would be able to vary based on label condition. (That said, I would not expect start-point to vary with label validity since the mapping of validity to left/right is random.)

Response 4:
We also agree with this point. In response, we fixed starting point across valid and invalid label conditions within experiments. The results showed that the starting points in all studies revealed a small, but statistically significant, bias to detect objects on the right side of the central border. **We now report that finding (see pp 25-26).** The right-side bias did not vary with study, however (Fig 6, third row, right). Given that objects were depicted equally often on the left and right sides of the displays, participants' slight, condition independent, preference to detect objects on the right side may reflect a bias to press the right button when their attention lapsed (see also page 26).

Point 5. One point of additional flexibility that the authors did not consider is whether non-decision time might vary based on label condition. Since the major theoretical ideas are based on faster (pre-decisional) activation of an object representation/semantic network, I would think it natural to see if some of the

benefits of valid labels might be reflected in faster non-decision times. (A heuristic to see if there might be empirical support for this would be to compare the fastest RTs across valid and invalid conditions—since the fastest RTs determine the positioning of non-decision time in the DDM, this provides a useful clue as to the necessity of freeing up non-decision time.)

Response 5:

Follow your recommendation, we compared the fastest response times (fastest 20%) between valid and invalid conditions within study 1 and 2 using a 2 (study: 1 vs. 2) x 2 (label: valid vs. invalid) mixed ANOVA. The results showed a main effect of label type: fastest RT is longer in the invalid condition than in the valid condition, $F(1, 206) = 99.42$, $p < 0.001$, $\eta 2 = 0.33$; and an interaction between label and study, $F(1, 206) = 4.58$, $p < 0.001$, $\eta 2 = 0.02$: the difference between valid and invalid RT is larger in study 2 than in study 1. Based on these results, we allowed non-decision time to vary with label condition. In both studies, we found that non-decision times were longer following invalid rather than valid labels, and in study 2, non-decision times on invalid label trials were statistically longer than non-decision times in the control study. (No differences from control were found in study 1.) We attribute these differences in non-decision times to lower response uncertainty on valid trials compared to invalid trials (See Hellmann et al., 2023) because of faster (pre-decisional) activation of the semantic network corresponding to the object depicted in the display.

Revised Part (P19):

Additionally, to determine whether valid labels lead to faster response times independent of evidence integration, we allowed for the possibility that labels can affect the non-decision time.

$$T_{er} = T_0 + T_l L \qquad (5)$$

Where T_0 denotes the baseline non-decision time and T_l is the change in non-decision time for a valid label (Again, $L = 0$ for invalid).

Revised Part (P26):

Non-decision time differences should be evident in the fastest responses. Therefore, to further examine T_{er}, we compared the fastest 20% of RTs between valid and invalid conditions in study 1 and 2 using a 2 (study: 1 vs. 2) x 2 (label: valid vs. invalid) mixed ANOVA. The results showed a main effect of label type: the fastest RTs are longer in the invalid condition than the valid condition, $F(1, 206) = 99.42$, $p < 0.001$, $\eta 2 = 0.33$ (see

see fig S5 in supplementary material_2); and an interaction between label type and study, $F(1, 206) = 4.58$, $p < 0.001$, $\eta 2 = 0.02$: the difference between valid and invalid RTs is larger in study 2 (-0.068 s) than in study 1 (-0.044 s). Hence, the analysis of fastest RTs supports the T_{er} results returned by the DDM.

Revised Part (supplementary material_2):

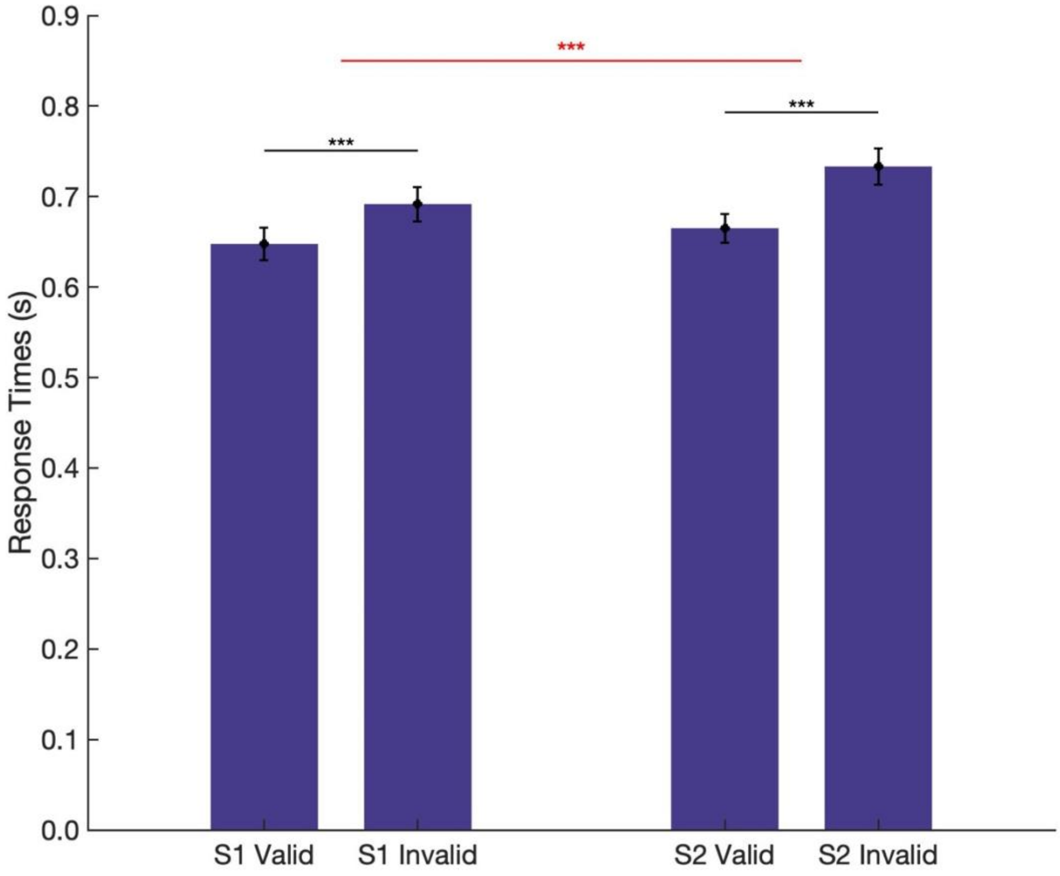

Fig S5 Comparison of reaction times between fastest 20% valid and invalid trials. *** indicates p < 0.001. Red asterisks represent interaction between label type and study.

Point 6. The description of the scaling property of the DDM is, I think, slightly inaccurate. The parameters of the DDM are only identified on a ratio scale, which means that one parameter must be chosen to be fixed to an arbitrary value. The authors are correct in pointing out that the diffusion

coefficient is usually chosen as the “scaling parameter,” but this is an essential feature of the model, rather than a means of simplifying it. (Apologies if this was the intent of this section and I failed to recognize it! I see that this is explicitly mentioned later on page 16.)

Response 6:

Thanks for this useful comment. The original description was indeed problematic. We have rephrased the description of the noise parameter.

Revised Part (P18):

Because the parameters of the drift diffusion model are only identifiable up to a scale factor (Bogacz et al., 2006), the noise parameter (s) is typically set to 1.

Point 7. – Parameter estimation – I am glad that code have been made available on the OSF, but I think more explicit detail about parameter estimation needs to be provided in text. Was MLE done for individual trials or was it done using a quantile-based approach?

Response 7:

We fit the DDM to behavior from the Skocypec and Peterson (2022) studies using a maximum likelihood approach on individual trails. We have provided more details of the how the MLE was computed in the paper.

Revised Part (P20):

We fit the DDM to behavior from the Skocypec and Peterson (2022) studies using a maximum likelihood approach. In particular, we find the parameter values that maximize the log likelihood of observing the choices and response times on all trials $i = 1, \dots, N$.

$$LL(\theta) = \sum_{i=1}^{N} \ln L_i(\theta) \quad (6)$$

where the individual trial likelihood is of observing choice C_i made after response time T_i is:

$$L_i(\theta) = f_{ddm}(C_i, T_i | \theta) \quad (7)$$

Where θ denotes the parameters of the model and f_{ddm} is the first passage time density computed using the method proposed by Navarro and Fuss (2009). TheheheheheheThe MLE procedure was run in MATLAB by minimizing the negative log-likelihood function using the *fmincon* function. Data and code to replicate these analyses are available at https://osf.io/g3mxb/.

Point 8. Model fit – The authors provide a visualization of model fit in Figure 5, but the figure is

somewhat difficult to read because it requires aligning spatially separated bars. A better visualization would use something like floating symbols overlaid on top of the bars to display model predictions.

Response 8:

We have changed the layout of figure 5. Now, simulation and actual data easier to compare because they are adjacent.

Revised Part (P21):

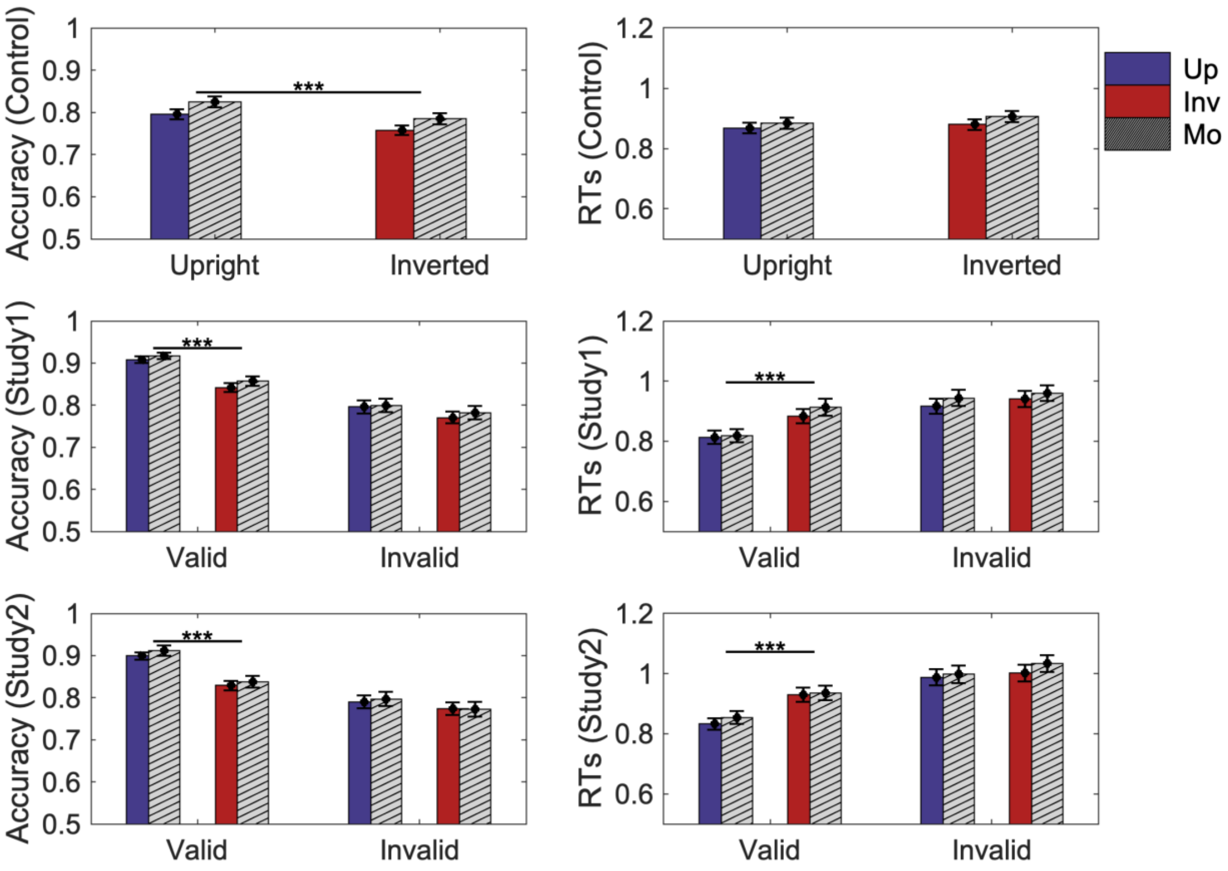

Fig 5 Accuracy (column 1) and response times (RTs) in seconds (column 2) for both experimental data and data simulated from the model for both control (row 1) and experimental studies 1 and 2 (rows 2 & 3, respectively). Blue bars and legend "Up" represent the upright condition. Red bars and legend "Inv" represent the inverted condition. Filled bars represent Skocypec & Peterson's (2022) behavioral data; striped bars and legend "Mo" represent data generated from the analytical expression of the model. Error bars represent pooled standard errors. *** indicates $p < 0.001$.

Point 9. Extending on this, it would be nice to see both the data and model predictions for entire RT

distributions for correct and error responses using something like a quantile probability plot. I can see that the RT predictions of the model seem to be a little off (e.g., for invalidly labelled inverted stimuli), but presumably these are all showing mean RTs (the figure doesn't specify) or some other central tendency measure. One question is whether the model predictions are off for (say) the mode of the RT distributions or if the mean is being pulled up by misses in the extreme tails.

Response 9:

We appreciate your suggestion to more closely examine the correspondence between model and data over the entire response time (RT) distribution. To this end we create two new Figures. The first is a quantile-quantile plot for response times for error and correct trials (in the paper as Fig S3, pasted below). This plot demonstrates the close correspondence between the model and the data over the whole range of response times. Indeed, an independent t test between model and data shows no significant difference between the response times for any quantile (*p*s > 0.07). In addition, this Figure highlights the source of the slight overestimation of data response times by the model illustrated in Fig 5 (see p. 9 of this document for Fig 5 which is shown in the response above): The model tends to slightly, albeit insignificantly, overestimate the longest response times on correct trials.

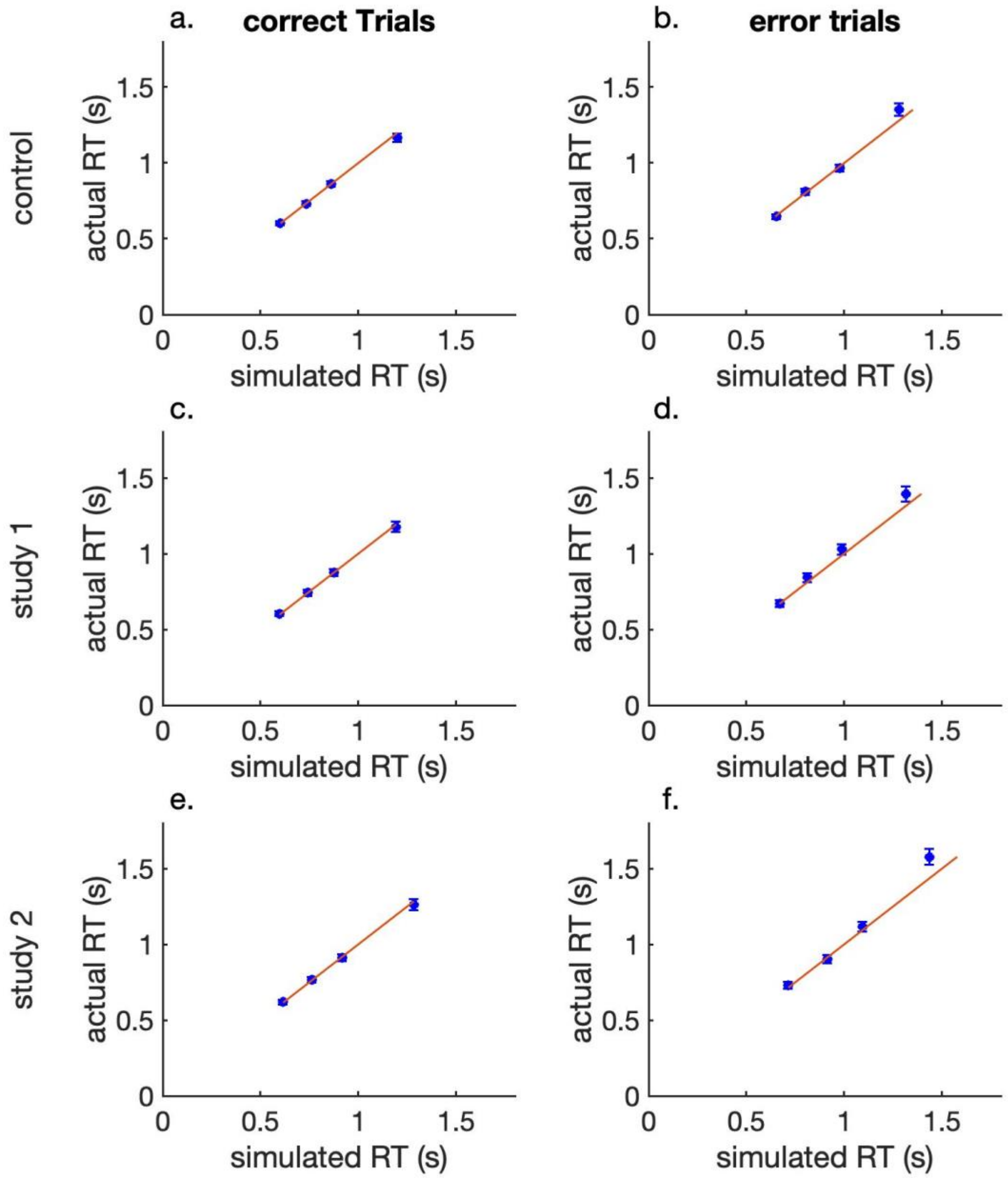

Fig S3 Quantile-quantile plot for control experiment. Left column is for correct trials, and right column is for error trials. First row represents control group, the middle row is study 1, and the bottom row is for study 2. Quantile levels are 24.5%, 49%, 73.5%, 98%, having an ascending order from the left to the right side, the final 2% trials are excluded. The reaction time of each quantile is computed for each participant first and then averaged over all participant. Error bar is pooled standard error.

The second new Figure (Fig S4, pasted below) compares the accuracy of the model and the data across the range of response times for the three studies. While some conditions showed differences in the fourth quantile (indicated by black asterisks in Fig S4), the majority of simulations aligned closely with the actual data.

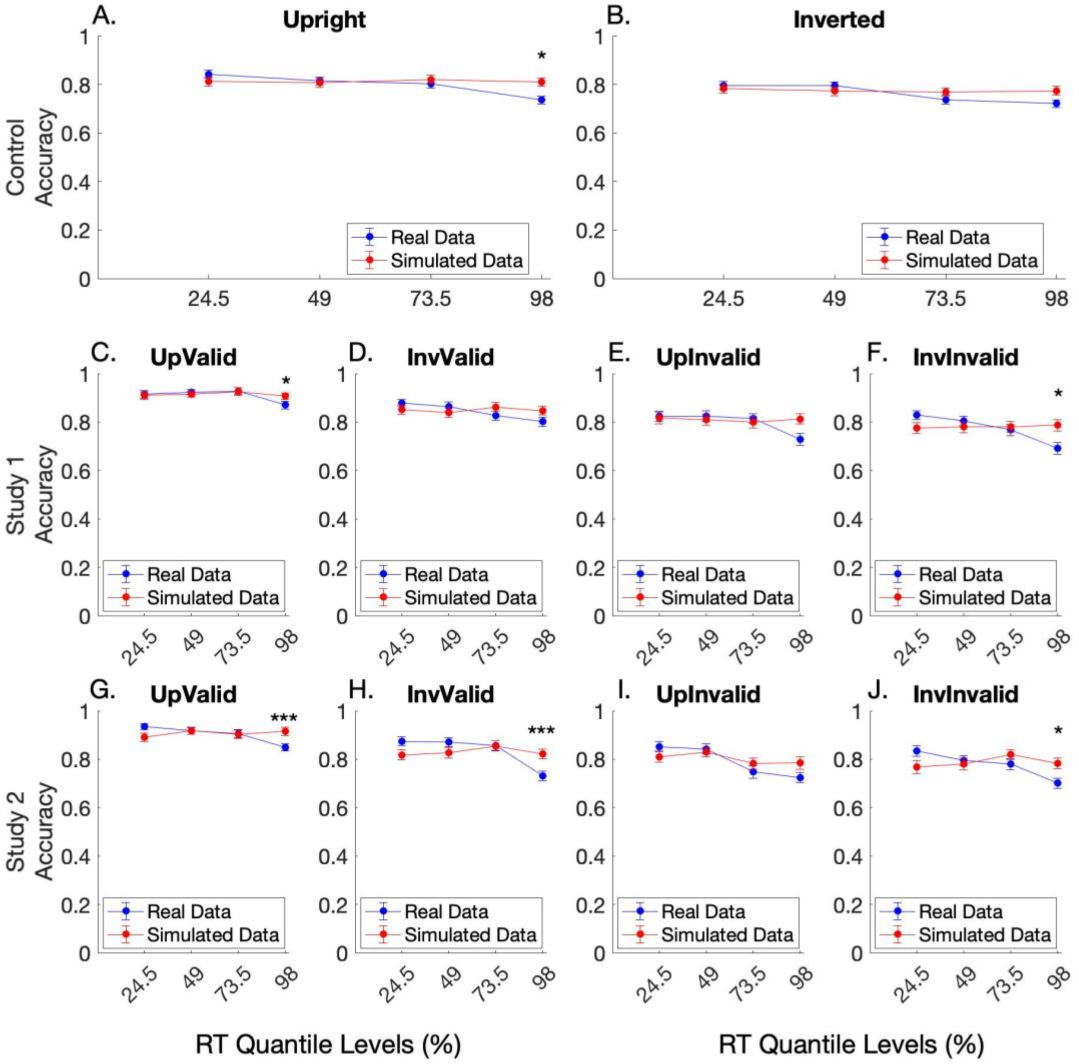

Fig S4. Accuracy against reaction time for actual data and simulated data for four quantiles. Quantile levels are 24.5%, 49%, 73.5%, 98%. Averaged accuracy was computed on each quantile for each participant and then averaged over all participants. First row represents control group, the middle row is study 1, and the bottom row is for study 2. In each axis, red line represents simulation, and blue line represents actual data. Error bar is pooled standard error. Black asterisks indicate differences between the actual data and the simulated data at the corresponding quantile levels represented along the x-axis. *** indicates $p < 0.00025$, * indicates $p < 0.0125$ after Bonferroni correction. The figure showed a good fit of the model to the data, especially in the first 3 quantile of the data.

Through these two new Figures, we find the slight overestimation of response times and accuracy shown in Fig 5 is mainly caused by responses in the last quantile. We consider two possible explanations for this discrepancy: 1) **Model Limitations:** The model is too limited to capture additional cognitive process such as lapses in attention. 2) **Speed-Accuracy Trade-Offs:** Participants might collapse their decision thresholds dynamically. On the other hand, despite its limitations, the present model effectively captures the overall decision-making process, especially within the first three quantiles of the data. We have expanded on these points in the revised manuscript, specifically in the "Limitations of This Research" section.

Revised Part (P34):

One limitation of the current research is that our model (DDM) tends to overestimate RTs and ACC in the last quantile (albeit slightly). As shown in the quantile-quantile plot in the supplementary material Fig S3, this discrepancy was mainly caused by the last quantile of correct trials. For this discrepancy, we consider 2 possibilities, 1) Model Limitations: The standard DDM assumes consistent decision-making processes across all trials. However, in the slowest correct trials, participants may engage in additional cognitive processes not captured by the model, such as increased deliberation or occasional lapses in attention that are subsequently corrected before responding. 2) Speed-Accuracy Trade-Offs: Participants might collapse their decision thresholds dynamically. To understand how good the fit to the data by the model was, we first tested the RT deviation by comparing the simulated RT to the actual RT using independent t-tests for each quantile. We didn't find significant differences between simulation and actual RT, $ps > 0.07$. Next, as shown in Fig S4, we plotted the accuracy against RT quantile for both simulation and actual data for all of the conditions in the three studies. For each condition, we used independent t tests to examine the difference between simulated and actual data. While some of the conditions showed a difference only in the 4th quantile (see black asterisk in Fig S4), most of the simulations are aligned with the actual data and none of the differences evident in studies 1 and 2 was larger than those evident in the control study, $ps > .24$. Therefore, we suggest that the model captures the overall decision-making process, especially the first 3 quantiles of the data.

Point 10. Another question is whether the RT data in these panels are combining correct and error responses or if only correct responses are being shown. (Apologies if I have missed this detail.)

Response 10:

The RT data shown in the paper combines correct and error responses. We have also stated this point in the paper.

Revised Part (P20):

As can be seen in Fig 5, after fitting, the drift diffusion model captures all the main effects in both the control and experimental studies. (All the reaction time shown in Fig 5 combine correct and error responses.)

Point 11. Interpretation of Parameter Estimates – This point relates to my concerns about model parameterization (Point #3). I think some of the drift rate effects are quite interesting and potentially important, but I am curious how strongly they were affected by also having freely varying thresholds and starting-points. Since all three of these parameters affect predictions for both accuracy and RT, it is difficult to discern how much stock to place in the drift rate results, for example. The ambiguity introduced by the way the authors parameterized the model has knock-on effects for inferences about individual parameters that make it difficult to evaluate the general theoretical conclusions.

Response 11:

We thank the reviewer for this valuable comment. As we discussed above (in response to your point 3), we re-ran our model with fixed starting points and thresholds across conditions within experiments. The same main effects were found in drift rates (i.e., orientation effect in valid condition).

Revised Part (P22-P24):

Drift rates obtained in the labels-present studies (studies 1 and 2) were first compared to drift rates obtained in the control labels-absent study to determine whether valid labels increase drift rate, invalid labels reduce drift rate, or both effects occur. Specifically, for each of the studies, a 2 (study: 1 [or 2] vs. control) x 2 (orientation: upright vs. inverted) mixed ANOVA was conducted for drift rates following valid and invalid labels separately. To preview: our drift diffusion model results show that valid labels increase drift rate whereas invalid labels did not affect drift rates.

Valid labels versus No Labels: In both study 1 and study 2, drift rates were significantly higher when valid labels preceded the displays compared to when no labels preceded the displays in the control study and the increase was larger for the upright than inverted displays (see Fig 6, top row). For both studies these effects were evident in main effects of orientation and study and an interaction between orientation and study. Drift rates were higher in the upright condition than the inverted condition: for study 1, $F(1, 216) = 104.72$, $p < 0.001$, $\eta 2 = 0.33$ and for study 2, $F(1, 214) = 105.95$, $p < 0.001$, $\eta 2 = 0.33$. Drift rates were higher in the labels-present studies than in the control study: for study 1, $F(1, 216) = 50.78$, $p < 0.001$, $\eta 2 = 0.19$ and for study 2, $F(1, 214) = 28.62$, $p < 0.001$, $\eta 2 = 0.12$. For both studies, the interaction between study and orientation reached significance, for study 1, $F(1, 216) = 28.65$, $p < 0.001$, $\eta 2 = 0.12$ and for study 2, $F(1, 214) = 29.53$, $p < 0.001$, $\eta 2 = 0.12$. Simple effects analyses indicated that the increase in drift rate following a valid label was larger in the upright than in the inverted condition: Control drift rates were 0.975 for upright displays, which is significantly higher than the 0.804 observed in inverted displays ($p = 0.001$). Study 1 mean increment upright = 0.825 (SD for the mean difference = 0.101, 95%CI [0.626, 1.024], $p < 0.001$); mean increment inverted = 0.451 (SD for the mean difference = 0.091, 95%CI [0.272, 0.630], $p < 0.001$). The same was true for study 2: mean increment upright = 0.657 (SD for the mean difference = 0.099, 95%CI [0.461, 0.853], $p < 0.001$); mean increment inverted = 0.275 (SD for the mean difference = 0.088, 95%CI [0.101, 0.449], $p = 0.002$). The evidence that drift rate is higher following valid labels in the labels-present studies than in the control labels-absent study and that the increase is greater for upright than inverted displays support the Semantic Network hypothesis rather than the Label-feedback hypothesis where orientation effects are not predicted.

Comparison of drift rates following valid labels in study 1 and study 2. To examine whether drift rates on valid label trials were affected by the context shaped by the invalid

labels shown on other trials in the labels-present studies, we conducted a 2 (study: 1 vs. 2) x 2 (orientation: upright vs. inverted) mixed ANOVA on valid drift rates. Drift rates following valid labels were higher in study 1 (M = 1.527) than in study 2 (M = 1.356), $F(1, 206) = 4.03$, $p = 0.046$, $\eta 2 = 0.02$ (see Fig 6, first row). This small, but statistically significant effect indicates that the rate of evidence accumulation following valid labels is affected by the context established by the type of invalid labels present on other trials within a study. In study 2, the semantic network of the target object overlaps with the semantic network of the object denoted by invalid labels because they are both in the same superordinate-level category. This overlap, present on 50% of the trials, may introduce enough noise in the evidence accumulation process to cause the reduction in drift rate observed on valid trials.

Invalid labels versus No Labels. Mixed ANOVAs comparing drift rates following invalid labels to those observed in the control labels-absent study were conducted separately for study 1 and 2. The factors were study ([1 or 2] vs. control) and orientation (upright vs. inverted). For both studies, this comparison revealed main effects of orientation: drift rates were higher when displays were upright rather than inverted (study 1: $F(1, 216) = 28.49$, $p < 0.001$, $\eta 2 = 0.12$; study 2: $F(1, 214) = 21.79$, $p < 0.001$, $\eta 2 = 0.09$). No interaction between study and orientation effect was observed in either study 1, $F(1, 216) = 0.20$, $p = 0.660$, or study 2, $F(1, 214) = 0.96$, $p = 0.329$. We also compared drift rates following invalid labels between study 1 and study 2 with a Mixed ANOVA, the results showed a main effect of orientation, $F(1, 216) = 13.73$, $p < 0.001$, $\eta 2 = 0.06$. No other significant effect was observed, $ps > 0.205$.

Reviewer #2: This paper uses drift diffusion modelling to investigate the mechanisms underlying a previously published, labelled object detection task. The Label-Feedback hypothesis is that the presence of a valid label pre-activates low level visual features which leads to faster object detection; however, the visual features pre-activated are invariant to orientation. Whereas, the semantic network hypothesis adds that in addition to priming, an invalid label can cause semantic interference depending on the degree of relatedness between the label and object. Also, low level visual features should be sensitive to the frequency of orientation because semantic networks would store that information. In terms of the model, if orientation matters, we expect to see a change in drift rate and, if there is semantic interference, we expect to see noise increase, which should lower drift rates and increase the threshold. The authors find evidence of both effects. The concluding argument, as I understood it, is that semantic interference entails semantic activation; therefore, object detection entails activation.

Point 1. The paper is well written. In particular, the modelling sections was easy to follow. I think how the computational model demonstrated the interference effect as noise is interesting; however, I think the paper would be stronger if they discussed how else the model might have captured the pattern of behaviour in the original study. Without the clear alternatives, I'm not sure the model could have captured the data any other way; in which case, the contribution of the paper is unclear as it simply reiterates the paper collecting the data.

Response 1:

We thank reviewer for their appreciation of our paper and our results, in particular, our finding that the semantic interference effect is best understood as noise. While we acknowledge that, under the current model, the effects in drift rate are anticipated, our research is the **first application of a Drift Diffusion Model (DDM) to understand the mechanisms of semantic influences on object detection:** We are the first to introduce the DDM to the study **the mechanisms of semantic influences on object detection**, providing a novel methodological approach to this phenomenon. Second, this paper makes a contribution by **providing a quantitative description for the first time:** This application allows us to offer a quantitative description of the perceptual decision-making process in figure-ground perception following word labels, which has not been previously achieved. Third, this paper provides n**ew insights into parameters T_{er} and a**: Importantly, our findings demonstrate that the model is sufficient to account for the data, which in turn makes it possible to analyze and estimate key parameters T_{er} and a.This approach marks the first time that parameters like T_{er} and a have been quantitatively described in this context. We have supplemented our previous discussion of the importance of the modeling effort on page 9.

Revised Part (P10):

Here, we investigate whether applying a Drift Diffusion Model (DDM) to figure-ground perception—for the first time—can capture Skocypec and Peterson's results. By doing this, we aim to provide a quantitative description of the orientation effect through drift rate analysis. Additionally, this novel application of modeling allows us, for the first time, to extract insights into key parameters such as non-decision time and boundary separation (i.e., threshold), deepening our understanding of the underlying cognitive processes. Lastly, we can also test whether there is any additional process that the model cannot identify, such as lapses of attention.

Point 2. In my reading, the key contribution of the paper is the demonstration of semantic interference as noise. I am not sure how much the orientation manipulation undermines the Label-Feedback hypothesis, which to my knowledge, is agnostic about the typical frequency of the orientation of the visual feature detectors and, thus, charitably could accommodate this result.

Response 2:

Thanks for appreciating the importance of our demonstration that semantic interference should be understood as noise. We don't believe that the Label-feedback hypothesis can accommodate the orientation effects. We discuss this in detail in the section " Label-Feedback Hypothesis versus the Semantic Network Hypothesis " starting on page 32.

Revised Part (P33):

Can the Label-Feedback hypothesis be altered to accommodate our results by supposing, for instance, that feature representations are orientation dependent? The classic view in perception research, supported by much research, is that visual features are orientation independent (e.g., Gayet, et al, 2014; Humphreys & Riddoch, 1984), whereas orientation dependence is a hallmark of configuration effects. What about supposing that the Label Feedback hypothesis extends to features in context? We don't see how this would help, as features in context are familiar configurations, which are not low-level representations. In the Label-Feedback hypothesis, feedback from high-level word representations extends to low-level features of objects (Lupyan & Ward, 2013). Moreover, in other experiments, we have found that that the same features arranged in

novel configurations do not operate as object priors, even when sub-configurations comprising familiar parts are intact (e.g., Flowers & Peterson, in review; Peterson, et al, 1991). Finally, our evidence contrary to the Label-Feedback hypothesis extends beyond orientation dependency: Drift rates were lower, and thresholds were higher when labels denoted an object in the same, rather than a different, superordinate-level category as the object in the display. Indeed, in study 1, invalid labels denoting objects in a different superordinate-level category had no effect on either drift rate or thresholds. On the Label-Feedback Hypothesis, the opposite pattern should have been observed because the features of objects in a different superordinate-level category differ more from the features of the objects in the display, and hence, would require more prediction revision which would take longer. These considerations suggest that the Label-Feedback Hypothesis cannot easily be altered to account for our results. The pattern of results is better fit by the Semantic Network Theory of Label effects.

Point 3. Finally, I'm not sure the concluding argument logically follows. While it's clear that introducing an expectation via a label allows semantic activation to influence object detection. No evidence is provided that this semantic activation is entailed for object detection without such expectations.

Response 3:

Our claim that semantic activation is entailed for object detection without expectations induced by labels relied on a finding we reported in the initial submission that thresholds were higher following valid labels in study 1 than they were on control trials where labels were absent. Following suggestions both you and Reviewer 1 made, in the revision we determined thresholds for each study rather than for the label types within a study. When we used this method, the thresholds in study 1 and the control study did not differ significantly. Therefore, we have removed our claim that semantic activation is entailed for object detection without expectations induced by label type.

Point 4. As a technical note, I would have appreciated more details on the parameter recovery analyses, specifically what was the decision rule to suggest that something is recovered and why is that an appropriate decision rule. Some of the correlations are rather small and even for some higher correlations, the scale is off by a wide margin. Why is the scale not important? In light of the importance of noise in

the interpretation of the semantic interference results, I think it would be important to illustrate how fixing the scale of the noise changes the other parameters (e.g., as a graph like Figure 3).

Response 4:

Thanks for this valuable comment. After updating our model, we have also redone our parameter recovery and updated Fig S1 and S2 in the supplementary materials. The current plot shows a good correlation among all parameters. We also fixed the scale on the figures.

Revised Part (supplementary materials_1**):**

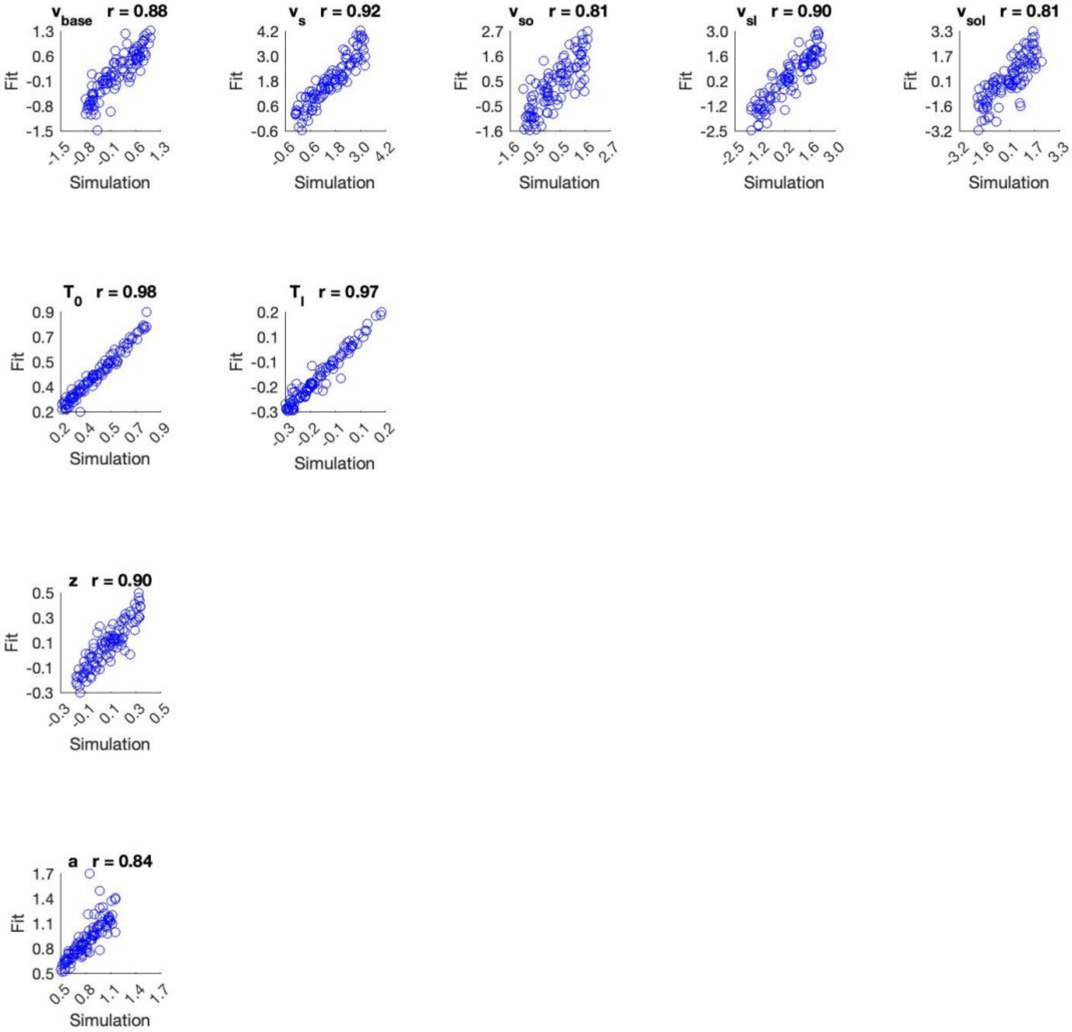

Fig S1 Parameter recovery for the full parameter model of the Experimental conditions with MLE fits. The value on the x axis represents simulation value, and value on y axis represents the value for the fitting process. Each row in the figure represents the components of up to 4 different parameters. Values of r on top of each axis represent the correlation coefficient. Note the scale of the y axis changes across graphs.

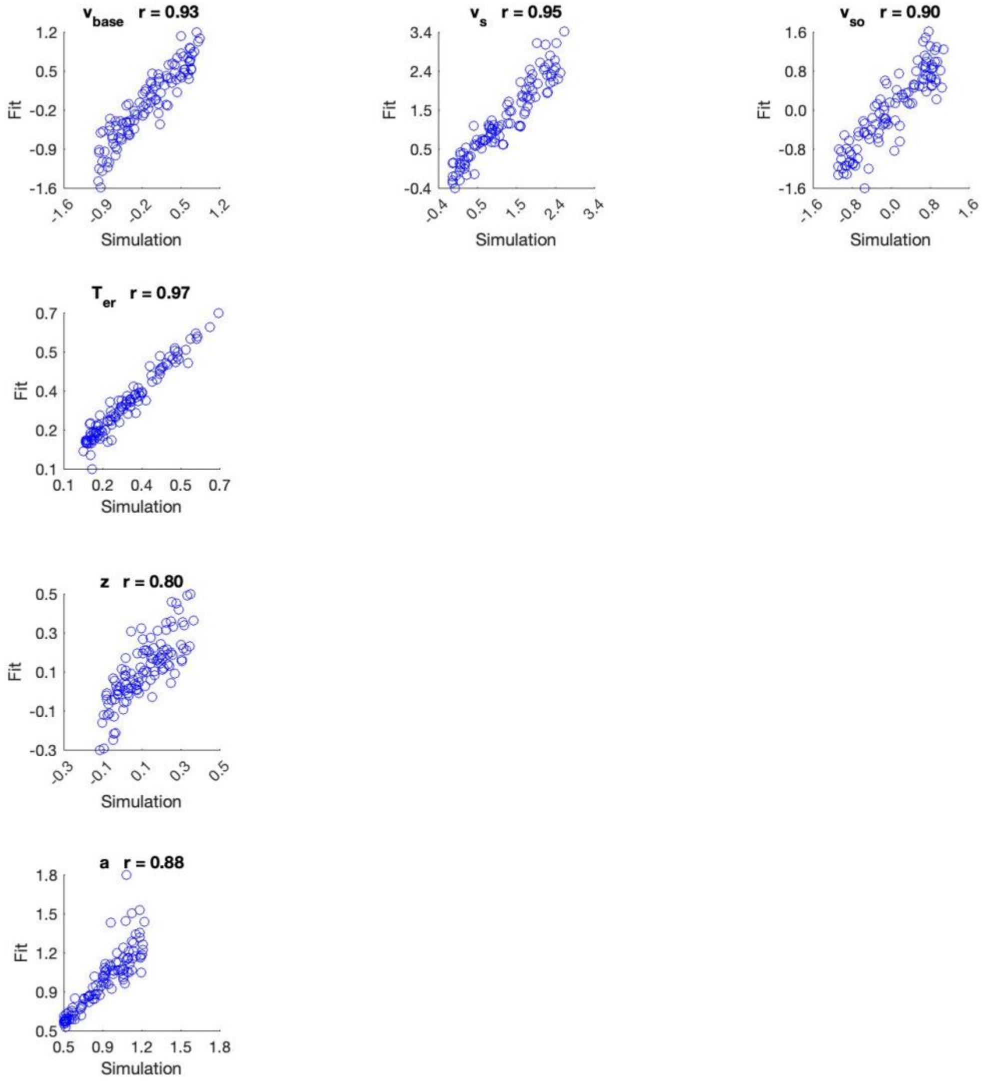

Fig S2 Parameter recovery for the model of the control conditions with MLE fits. Parameter recovery with MLE fits for the full parameter model. Axis and values of r have the same meaning as in Fig 1, the only difference is that we have 6 instead of 9 parameters for the control group.
