# Supplementary material for: Semantic influences on object detection: Drift diffusion modeling provides insights regarding mechanism
Source: PLoS Comput Biol. 2025 Jun 11;21(6):e1012269. doi: 10.1371/journal.pcbi.1012269 (PMC12194206; doi:10.1371/journal.pcbi.1012269)
Supplement: Attachment — Submitted filename: Response to Reviewers.pdf [file pcbi.1012269.s007.pdf]

Response to Reviewers

We greatly appreciate the thoughtful comments and suggestions of the reviewer. In what follows we note each of the comments in blue ink and describe our response in black ink. The revised part from the manuscript is in red ink. Our manuscript is further improved after making the additional changes recommended by the reviewer.

Reviewer 1:

Point 1. Thresholds should be compared across Studies 1 and 2. Currently, threshold parameters for Studies 1 and 2 are compared against thresholds in a no-label control. This is fine to establish that semantic activation is involved in the detection process but it would be more instructive to know how threshold is affected by the degree of activation. Indeed, based on the current results, one would expect to see a threshold difference between Studies 1 and 2 if there is no semantic overlap between targets and invalid labels in Study 1. I note, however, that it would be perfectly understandable if the difference across Studies fails to reach significance—there is likely some low level of activation achieved even by labels for different semantic categories—but it would be instructive to check for.

Another reason for performing a direct comparison of Studies 1 and 2 is that the authors comment on the current results as if a direct study-to-study comparison had been made. For example, on page 33, it is mentioned, "…thresholds were higher when labels denoted an object in the same, rather than a different, superordinate-level category as the object in the display." This claim can only be supported by comparing thresholds across studies directly.

Response 1:

We appreciate the suggestion to directly compare threshold across Studies 1 and 2. We performed this comparison but the threshold difference between the two studies was not significant, $p = 0.140$. It is possible that a small amount of semantic activation occurs even in Study 1, or that our data did not provide sufficient power to detect a subtle difference between Studies 1 and 2. We have modified our statement in the original text and also discussed the direct comparison between studies 1 and 2.

Revised Part (P29):

> Thresholds were higher than control only when the label denoted an object from the same superordinate-level category as the displayed object, not when it denoted an object from a different category.

Revised Part (P29):

We note, however, that an independent t-test comparing thresholds between study 1 and study 2 showed no difference, $p = 0.140$. Future research is needed to investigate if a small amount of semantic interference occurs even in Study 1.

Point 2. Are "noise-dependent" changes in threshold strategic or under top-down control?
The argument (e.g., page 32) that additional noise due to semantic overlap of invalid labels and objects increases decision thresholds is an interesting one. The current description of the nature of this effect strikes me as agnostic about the degree to which this is under strategic control (or otherwise influenced by top-down goals). My own view of these kinds of effects is that they likely reflect a strategic adaptation to the task environment, not unlike how changes in response bias can be induced by varying payoffs or the proportion of responses of a certain type required by the task. To the extent that threshold changes adapt to the presence of noise, one could in principle analyze changes in threshold over the course of the task (e.g., by fitting to model to successive blocks/trial epochs). I am not suggesting this can be done for the current study but this could be worth mentioning as a future research direction (e.g., likely requiring a hierarchical approach to model fitting).

Response 2:

Our current results explain the increased threshold in study 2 as a response to noise introduced by semantic overlap. However, we agree that this effect might not be purely automatic but could reflect top-down control. We added this point into the text.

Revised Part (P27):

An interesting question is whether threshold adjustments are a purely bottom-up response to increased noise or reflect top-down control. Future research can adopt more complicated models to investigate this question.

Point 3. Page 13 – "…we can fit the four free parameters of the model…" would be clearer if you said, "…we can estimate the four free parameters of the model…"

Response 3:

We have revised this sentence and now it is " we can therefore estimate the four free parameters of the model ".

Point 4. Page 22 – The in-text description of the drift rate increments for Studies 1 and 2 is difficult to parse. Consider presenting this information in a Table for better readability.

Response 4:

Thanks for this comment, we realize that it was confusing. We removed the descriptions in the text and added Table 1 showing drift rate increments over control for studies 1 and 2. The entire section now is much easier to read.

Revised Part (P19-P20):

To determine whether valid labels increase drift rate, invalid labels reduce drift rate, or both effects occur, drift rates obtained in the labels-present studies (studies 1 and 2) were first compared to drift rates obtained in the control labels-absent study. Control drift rates were 0.975 and 0.804 for upright and inverted displays, respectively, a statistically significant difference, $p = 0.001$. For each of the studies, a 2 (study: 1 [or 2] vs. control) x 2 (orientation: upright vs. inverted) mixed ANOVA was conducted for drift rates following valid and invalid labels separately. To preview: our drift diffusion model results show that valid labels increase drift rate over control whereas invalid labels did not affect drift rates. Figure 6 shows the drift rates obtained in the labels present studies; Table 1 shows the increment over control.

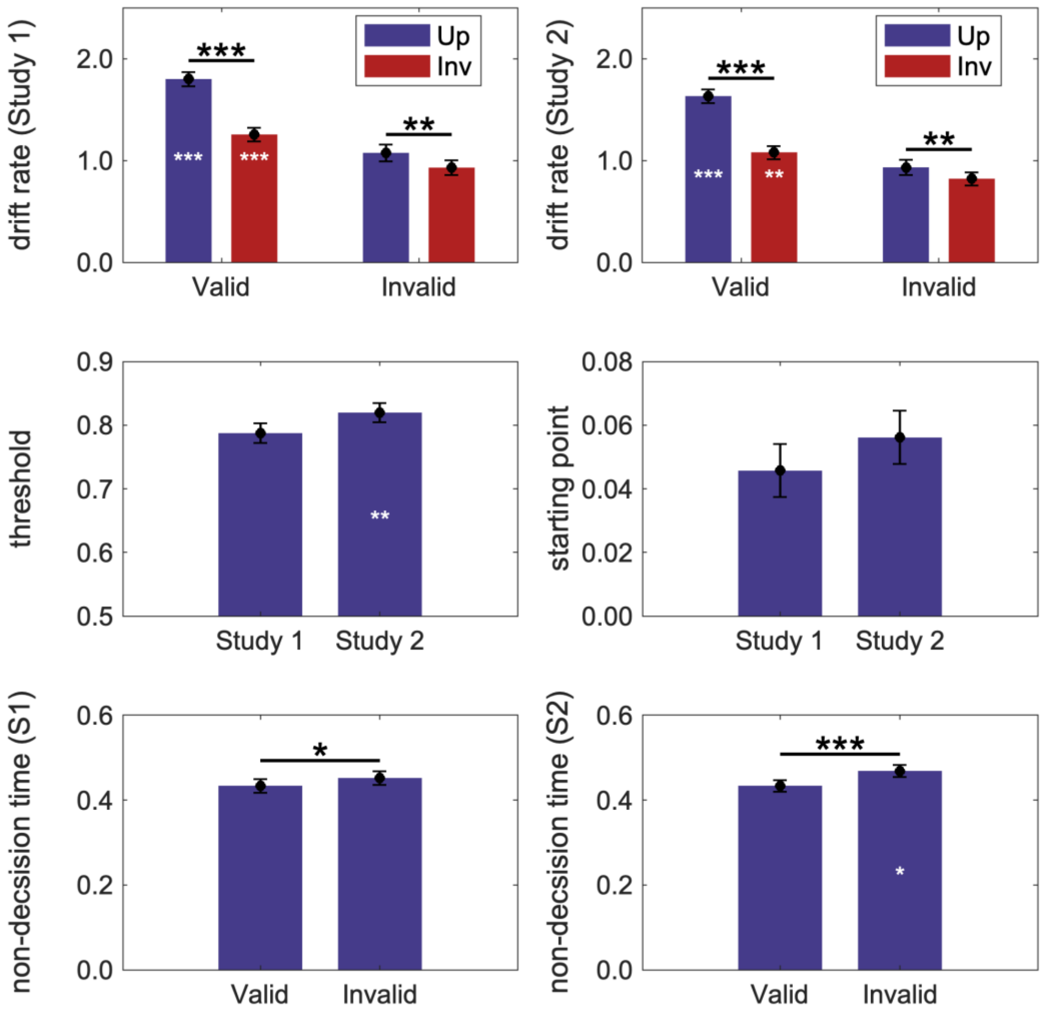

Fig 6 Model parameters in labels-present studies. (First row) drift rate (v). (Second row left) threshold (a). (Second row right) starting point (z). Note the different scales on the ordinates of the figures in the second row. (Third row) non-decision time (T_{er}). Higher values indicate a greater drift rate/threshold/starting point/non-decision time. For starting point, positive values indicate starting points toward the right side, negative values indicate starting points toward the left side. (Left column) study 1; (Right Column) study 2. White asterisks indicate statistically significant increments over control. Error bars represent pooled standard errors. *** = $p < 0.001$, ** = $p < 0.01$, and * = $p < 0.05$.

Table 1 Increments of drift rates in studies 1 and 2 over control

Study	*Orientation*	*Increment*	*95% CI*	*p-value*
Study 1	Upright	.825 (.101)	[0.626, 1.024]	< 0.001
	Inverted	.451(.091)	[0.272, 0.630]	< 0.001
Study 2	Upright	.657 (.099)	[0.461, 0.853]	< 0.001
	Inverted	.275 (.088)	[0.101, 0.449]	0.002

Note: Mean increment over control by condition in studies 1 and 2 (standard deviations in parenthesis), confidence intervals (CI), and *p*-values for the comparisons to control.

Valid labels versus No Labels: In both study 1 and study 2, drift rates were significantly higher when valid labels preceded the displays compared to when no labels preceded the displays in the control study (See Table 1) and the increase was larger for the upright than inverted displays (see Fig 6, top row). For both studies these effects were evident in main effects of orientation and study and an interaction between orientation and study. Drift rates were higher in the upright condition than the inverted condition (study 1: $F(1, 216) = 104.72$, $p < 0.001$, $\eta 2 = 0.33$; study 2: $F(1, 214) = 105.95$, $p < 0.001$, $\eta 2 = 0.33$). Drift rates were higher in the labels-present studies than in the control study (study 1: $F(1, 216) = 50.78$, $p < 0.001$, $\eta 2 = 0.19$; study 2: $F(1, 214) = 28.62$, $p < 0.001$, $\eta 2 = 0.12$). For both studies 1 and 2, the interaction between study and orientation reached significance (study 1: $F(1, 216) = 28.65$, $p < 0.001$, $\eta 2 = 0.12$; study 2: $F(1, 214) = 29.53$, $p < 0.001$, $\eta 2 = 0.12$). Simple effects analyses indicated that the increment in drift rate following a valid label was larger in the upright than in the inverted condition, for study1, $t(104) = 6.07$, $p < 0.001$; for study2, $t(102) = 6.12$, $p < 0.001$.The evidence that following valid labels the increment in drift rate over the control condition is greater for upright than inverted displays supports the Semantic Network hypothesis rather than the Label-feedback hypothesis where orientation effects are not predicted.

Point 5. Page 22 – I think the argument that the data cohere with the semantic network hypothesis better than with the label feedback hypothesis is convincing, but I think the claim about "…more prediction revision … would take longer" needs further clarification as to what is taking longer. I can understand behaviorally that responses will be slower due to (say) increased interference predicted by the label feedback hypothesis, but no explicit cause of what would make things take longer is currently mentioned.

Response 5:

We have revised the relevant section (p. 35) to clarify what we mean by processes "taking longer" under the Label-Feedback Hypothesis. Specifically, we now specify that, under this hypothesis, a larger discrepancy between the features of an invalid label and the display would necessitate greater prediction revision, and this internal revision process is expected to be time-consuming.

Revised Part (P29):

Indeed, in study 1, invalid labels denoting objects in a different superordinate-level category had no effect on either drift rate or thresholds. On the Label-Feedback Hypothesis, the opposite pattern should have been observed because the features of objects in a different superordinate-level category differ more from the features of the objects in the display. Under this hypothesis, a larger discrepancy would require more prediction revision; updating predictions would take time and would prolong internal processing, therefore lowering drift rates or elevating thresholds. The absence of these predicted effects suggests that the Label-Feedback Hypothesis cannot easily be modified to account for our results. The pattern of results is better fit by the Semantic Network Theory of Label effects.
